# Grief experience among ICU staff with loss of family members during COVID-19 outbreak in IRAN: A qualitative study

**DOI:** 10.3389/fpsyt.2022.904760

**Published:** 2022-07-18

**Authors:** Shabnam Nohesara, Mahdieh Saeidi, Hesam Mosavari, Leila Ghalichi, Mahmoud Reza Alebouyeh

**Affiliations:** ^1^Department of Psychiatry, School of Medicine, Mental Health Research Center, Psychosocial Health Research Institute (PHRI), Iran University of Medical Sciences, Tehran, Iran; ^2^Research Center for Addiction and Risky Behaviors, Iran University of Medical Sciences, Tehran, Iran; ^3^Student Research Committee, School of Medicine, Iran University of Medical Sciences, Tehran, Iran; ^4^Mental Health Research Center, Psychosocial Health Research Institute (PHRI), Iran University of Medical Sciences, Tehran, Iran; ^5^Department of Anesthesiology, School of Medicine, Iran University of Medical Sciences, Tehran, Iran

**Keywords:** COVID-19, grief, experience, ICU staff, qualitative study

## Abstract

**Introduction:**

The COVID-19 crisis created a lot of problems in people's lives. Different lifestyles, mental health, communication, rituals and traditions, particularly those involved in mourning, have changed drastically. Medical staff faced numerous critically ill patients every day. This greatly distressed the staff, especially the ICU staff. The end result was considerable amounts of mental distress for the medical staff who lost family members to COVID-19 making the distress even more complex.

**Methods:**

We carried out this qualitative research to study the grief experiences of 12 Iranian ICU staff members at the Rasoul Akram Hospital who had experienced the loss of a family member to the COVID-19 pandemic. We studied the effects of how their own grief experience and how constant exposure to critically ill patients influenced their work with patients. All semi-structured interviews were held in the presence of a faculty member of the psychiatry department of Iran University of Medical Sciences. The interview on the grief experience among ICU staff during the COVID-19 pandemic, consists of 4 issues: Familiarity, Experience during the COVID-19 pandemic, Grieving the loss of a family member and Effects of parallel grief.

**Results:**

We found five common themes in the result of the experiences of the participants based on content analysis. These consisted of: complex grieving process, new experiences for coping with loss, more empathy for patients, change the meaning of death, and the need for support in work places. Likewise, there were 22 sub themes.

**Conclusion:**

Paying attention to the details of staff members' life, gender differences, and cultural aspects can give us a better understanding and perception of their grief experiences. This understanding brings out valuable points which can help policy makers pass better laws for the wellbeing of society and people in order to promote leadership in turbulent times.

## Introduction

The COVID-19 pandemic is a tragedy that caused a partial collapse of many social, economic, and health delivery systems worldwide. The world faced a pandemic that spread rapidly amongst people and took the lives of millions all around the world (1). Millions of people who lost their lives began their sad journey through hospitals and medical facilities that were miserably inadequate to handle the tidal wave of COVID patients overwhelming medical systems in all countries. Many physicians and nurses themselves lost their lives treating their COVID patients or lost their own family members without any opportunity to mourn their loved ones. The medical staff faced vast numbers of patients every day who were dying from COVID-19 (2, 3).

On the other hand, there was a lack of proper healthcare facilities in many medical centers lacking appropriate mental health care toolkits. This greatly distressed the medical staff, for they were terrified of transmitting the infection from the work area into their homes. The result was great mental distress for the medical staff who had to experience new and different coping strategies in a pandemic (4).

The pressure of working in the intensive care unit (ICU) brought on immense distress and a sense of acute responsibility with it. The pressure was added to by a sense of duty, changes to work protocols, the absence of loved ones, and the trauma of death all around (5).

Much research has shown that exposure to this pandemic has raised the risk of complicated grief caused by the fact that this infectious disease has been unknown and unpredictable, a lack of specific and efficient treatment to control and eradicate it, the continuity of the quarantine, the lack of the possibility to hold mourning rituals or funerals, the lack of the opportunity to be present in the ceremonies, strict laws of transportation, the mortality and morbidity of the virus, ICU staff's work environment that sometimes caused the risk of spreading the virus and even multiple fatalities in a family cluster in a short period (6–8).

Not being allowed to enter and stay with the patients of COVID-19 in hospitals and not being able to see them and say their last goodbye to their loved ones (9, 10) all raised the risk of distress and caused mental illnesses such as anxiety, depression, sleep disturbance, post-traumatic stress disorder (PTSD) and other medical conditions which raised the rate of suicidal attempts and affected the quality of people's lives (11, 12).

The medical staff, especially those who function as frontline workers in the ICU, were not only losing their own patients in their professional life but, like the rest of the society, were losing their loved ones in their personal life. Therefore, their experiences of losing their loved ones in these times changed. To attend to health protocols and keep social distancing, ceremonies and mourning rituals were canceled or held with very few people. Mourning rituals differ according to culture, but the common element is that these rituals are ways to calm the person down and relieve the person in mourning (13). The duration of the ceremonies was also cut short, and those in mourning were deprived of performing the rituals and had to go through their time of mourning slowly, sometimes facing huge complications and complexities (14). The grief and sorrow of the ones left behind were not acknowledged by the societies and relatives and these people were not able to process their grief and it was left unprocessed (15).

ICU staff is facing the daily influx of critically ill patients. Seeing and caring for people at the end stage of life has a heavy emotional toll which is what ICU staff face. The lack of sufficient personal facilities, safe diagnostic tests, effective curative methods, and the uncertainty of laws concerning quarantine regulations added to the anxiety and stress of ICU personnel (16).

Apart from the professional roles, these people also played essential roles in their personal lives that involved deep emotional attachments with family members. Therefore, they also faced pressure with worries about their family's safety and health (17). COVID-19 changed ICU staff's sleep cycles which can create severe emotional and mental pressure. Seventy-five percent of ICU staff faced sleep disorders, 85% faced medium to high stress, and 61% faced both (18, 19). The assessments show that many frontline health workers also faced the death of their own loved ones to COVID-19. Some reports claim that 51% of ICU staff experienced burnout during the pandemic. These people experienced various mental disorders and complicated grief because at times they were unable to be present at the death bed of their loved ones due to work pressures. They were deprived of the right to bid their loved ones farewell. They faced the unpredictable death of loved ones while they were also worried of such things as infecting family members. Knowing of the symptoms of the illness and its progression, prognosis and treatment can, on the one hand, positively influence them for they will have a realistic attitude on the illness and death while, on the other hand, it can create a sense of frustration and powerlessness to be of any help (20).

Some members of the medical team faced unique conflicts in confronting the death of their loved ones. Working in a place where they constantly meet critically ill patients while losing a family member to COVID-19 can have different effects on the way they deal with their grief. Facing patients in the work environment after the death of their loved ones desensitized some staff members and allowed them to deal more easily with the death of a patient and, in some cases, allowed them to empathize more with the patient. It sometimes made them re-experience the loss of their own loved one (21).

Every culture has certain mourning rituals to help the surviving individuals work through their experiences of loss and anguish. Social networking also provides limited channels for processing a sense of grief and loss, particularly for those who have heavy workloads and only limited time to leave the hospital to participate in mourning ceremonies (22, 23). In Iranian culture, there are mourning ceremonies of various kinds to help individuals do grief work. People come together in the deceased person's home to give moral support to the grieved person and keep the memory of the departed alive. They read from religious texts and pray for the salvation of the deceased in the afterlife (24). The mourners are not expected to work and carry on with their everyday tasks for a while after the loss of a loved one. During this time, they are supported by their families and friends so that they can go through the process of grieving. However, in the COVID pandemic, the work's extreme pressure prevented many health workers from going through this process to cope with their grief over their loss. Their sorrow continues to remain unprocessed (7).

There are very few studies to examine the experience of the frontline health care professionals who have lost their loved ones without the opportunity to do grief work during this pandemic. This was the impetus behind our attempt to study this phenomenon through qualitative research.

We studied the effects of how the grief experience of the health care providers working in the COVID-ICU influenced their work with patients and how constant exposure to critically ill patients influenced their own grief experience. We tried to identify and examine the different experiences of health workers who had lost their loved ones while serving those hospitalized for the COVID-19 in the ICU. One of our aims was to raise attention to the plight of these health workers to help with policies that would decrease the pressure the medical staff is experiencing in such situations.

## Methodology

### Overview

A qualitative study was conducted on 12 men and women who worked in the ICU ward and the COVID-19 emergency ICU in the Rasoul Akram Hospital at the beginning of 2022 in Tehran, Iran.

### Participants

At first, we identified the frontline COVID-19 medical staff in the hospital who had also lost a loved one to COVID-19 and invited them to join our study. In line with the ethical guidelines for clinical research to protect volunteers from trauma or abuse and inform them of the purpose and methods of the study, we obtained their informed consent before the interview making sure that they wanted to participate in the study on their own volition. We also assured their complete anonymity and their right to leave the study at any time they wished.

### Data collection

For data gathering purposes, in this study individual semi-structured interviews were used in person in a safe and calm environment. From the 125 ICU personnel 22 had suffered the loss of a loved one and 12 participated in this study. The term “loved one” refers to immediate and secondary family members. Those who had lost a co-worker were not included in the study. At the beginning of the interview, the aim of the study and the way in which it would progress was fully explained to the interviewees. At the end of the study, they were asked to give their feedback in order to improve the process. It was also stated that in order to benefit best from the contents of the session, the sessions would be recorded. The interviewees were assured of the security and confidentiality of the sessions and of the fact that the records would be kept where only the interviewers and their aides would be allowed to listen to the contents. Once the sessions were recorded, they would be kept in a safe place where only the researchers on the cases and their aides could have access to them. All the interviews which were kept as audio files would be written out in full by the interviewer and aides, based on the content analysis, the data gathering would continue until we found no new data (25). In this way, data gathering would end upon the termination of the interview #12.

All the interviews were carried out by a psychiatrist on the medical staff of the university and an assistant which is why all the interviews go through the same process. Apart from gaining the necessary information and carrying out the semi-structured interview, the psychiatrist would pay attention to the non-verbal communication of the participants. In case of witnessing any emotional sensitivity when describing difficult experiences while grieving, the psychiatrist would give appropriate emotional responses and pay particular attention to their non-verbal communication, their body language and acknowledge their emotions and act in a supportive manner. Next a special time, separate from the time of the interview, would be allotted to delving into these emotions of the individual. The person's mental state would be evaluated and in case it was necessary to go into psycho-education to control the situation communicational pathways to receiving necessary mental health services would be set up as psychotherapy and psychiatric sessions. Those interviewed who wished could then benefit from mental health services as they would be referred to a psychiatrist.

Initially the interview would be carried out with open questions. Then, depending on the needs of the individual the rest of the interview would be carried out with attention paid to details. In order to facilitate the process, a guideline would be set up to explain the methodology in detail by the psychiatrist and his aides explaining the beginning and process of the interview. Issues critical to the study and predicted conflicts would all be written out in full and placed at the disposal of the interviewer.

### Interviews

Instructions on the interview on the grief experience among ICU staff during the COVID-19 pandemic, consists of 4 issues: familiarity, experience during the COVID-19 pandemic, grieving the loss of a family member and effects of parallel grief.

In the first issue “familiarity” the age and gender of the participants in the time and duration of their service in the intensive care or the COVID-19 ward were studied.

In the second issue, questions about their experiences during the COVID-19 pandemic and some open questions were asked. Examples of such questions are as follows:

How do you explain serving at a time of COVID-19? Can you estimate the number of patients whom you were responsible for? Can you estimate the number of your patients who died because of COVID-19? Do you remember your first patient who died of COVID-19?

The aim was to study burden and workload both pre and post COVID-19.

In the third issue, questions about grieving the loss of the loved one were asked. Examples of such questions are as follows:

Have any of the people close to you passed away from COVID-19? Who? Do you remember the date of their death? After losing your loved one to COVID-19, how did you feel? How did you grieve? Were you able to take some time off work? Did you have a ceremony?

In the fourth issue, items about the effects of parallel grief were assessed. Examples of such questions are as follows:

Did you continue taking care of COVID-19 patients after losing your loved one/ones? How did the grief experience influence your work with patients and how did constant exposure to critically ill patients influence your own grief experience?

### Analysis

The given data in this research includes notes written by the staff who took care of patients. These semi-structured interviews focused on the four fields explained above. Each of these reports was considered as a unit of analysis and after being read many times the main codes were brought to attention and then categorized and classified and the main structures were formed.

Data analysis was carried out using the MAXQDA software. This provides a suitable environment for the analysis of data and producing the themes and sub-themes in a qualitative research (26). We also pursued an interactive set of categories and aimed for the flexibility to add new categories throughout analysis.

Interviews were transcribed as soon as possible. After verbatim transcription of the interviews, the text was read carefully and then independently coded by two researchers. The process was repeated several times to ensure the complete and correct understanding of the concepts. Units of the text were coded and recoded during this process, and codes were later organized into categories and themes. At this stage, the research team discussed the codes, categories and themes until consensus was achieved. The final set of codes, categories and themes were agreed upon by all members of the research team. After 12 interviews, no further codes emerged and recruitment ceased.

### Trustworthiness

The transferability, credibility, and consistency of the data were certified based on the Guba and Lincoln criteria as an essential part of qualitative studies. These were considered through prolonged involvement in the subjects, external and peer check, and discussing any relevant literatures explaining the raw data recording process and providing an explanation for the coding and analysis processes (27, 28).

### Ethical considerations

Informed consent was obtained from all the participants. This research was approved by the ethics research committee of the Iran University of Medial Sciences with unique number IR.IUMS.REC.1400.1148 and protocol number 22923. All the names were deleted to respect the anonymity of the participants.

## Results

The participants were two anesthesiologists, four nurses, two paramedics, and two service workers. The experiences of the participants in this article are shown in five themes and twenty-two sub themes (Table 1).

**Table 1:** The list of themes and sub themes.

Complex grieving process	Feeling guilty for being the carrier Feeling guilty due to lack of treatment facilities and vaccinations Feeling guilty and regret for not having the chance to take care of the loved one because of work load Unprocessed feelings due to the uncompleted grief because of exposure to the rapid influx of critically ill patients The lack of emotional support in the work place Inability to express their emotional experience and sense of grief during loss Exposure to COVID-19 patients and its high mortality rate Not being able to hold funerals
New experiences for coping with loss	Keeping themselves busy to avoid annoying thoughts and memories of the deceased Taking time off Changing to Non-COVID-19 wards The role of religion and spirituality Receiving and asking support from co-workers, family members, hospital and society
More empathy for patients	The need to continue working in the COVID-19 ward More attention and kindness for patients More understanding of the family situation Spending more time caring for the patients to decrease the feeling of guilt
Change the meaning of death	Normalization of the concept of death and feeling apathetic Accepting death as an inevitable phenomenon and having less fear
The need for support in work places	The need to receive empathy from the work place at a time of grief The need to increase personal protective supplies in crises The need to reduce working hours

### First theme: Complex grieving process

This issue contains six sub-issues which are directed toward the factors which complicate the grieving process. The factors shown in this issue show that these people's jobs and the work pressure they have to tolerate have a great effect on their experiences.

#### Feeling guilty for being the carrier

Some of these people explained that they were terrified by the fact that the illness was mysterious, unknown and rapidly spreading, especially in the beginning of the pandemic and they were constantly worried that they might be the cause of the virus being spread in society and in their families. Later when they heard that one of their loved ones was sick, they felt guilty that their job might be the reason.

*35 year old /female:* ^*^Each time after our shifts were over and we had to go home we were worried that we might take home the disease from the hospital. What if I made others sick? I wouldn't be able to forgive myself.

*28 year old /female:* ^*^ *My loved one would not have died if I didn't work at a hospital*.

*45 year old/female: In the beginning of the pandemic, everyone was scared of us because they knew we were working in the COVID-19 ward. My mother worked at the hospital in the NICU, but they were terrified of us who worked in the hospital*.

#### Feeling guilty due to lack of treatment facilities and vaccinations

Many felt guilty for lacking the proper knowledge, skill, and tools, including vaccines, to prevent the demise of their patients. Some people regretted the lack of health facilities, especially in the beginning of the COVID-19 pandemic, especially those who had lost a loved one before the vaccinations started. They expressed their regret and that they wished the vaccinations had started sooner and everyone had the chance to use them.

*35 year old /female:* ^*^I think that if we had had vaccines sooner, this would not have happened to my uncle; my uncle who was not vaccinated passed away, he died innocently.

*48 year old /female:* ^*^ *In the beginning we were desperate that there might not be any vaccination at all, but when we heard that the world was controlling the disease by getting vaccinated, we were heartbroken that the vaccine didn't come sooner and we had to watch our loved ones pass away*.

#### Feeling guilty and regret for not having the chance to take care of the loved one because of work load

Since we did not have a large enough work force and that there was an overflow of people coming into the hospitals, there wasn't time for the staff to take off from work and this caused less quality time with family, especially those families who lived in other cities. This caused a feeling of guilt in the staff and they felt that they didn't have much role in taking care of their loved ones.

*28 year old /female:* ^*^If I could have taken time off work for a while and go to my home town to be with my father, perhaps I could have spent the last days of his life next to him. I regret the fact that I didn't have this opportunity.

*45 year old /male:* ^*^ *In the beginning of the pandemic, we were not allowed to take time off work, our co-workers were affected by COVID-19 and were quarantined. I wanted to take time off work to stay with my family until they got well***.**

#### Unprocessed feelings due to the uncompleted grief because of exposure to the rapid influx of critically ill patients

Since the people we had interviewed constantly had to face critically ill patients and daily deaths, there was no time to process the grief they were experiencing to allow their emotions to be digested. These feelings accumulated. The interviewees reported the experience of people dying in front of them every day with no opportunity to talk about their feelings with anyone. In some shifts, they faced one patient after another dying with no time even to eat lunch or dinner.

*35 year old /female:* ^*^ *Imagine that a number of people would die every day in front of your eyes. In some shifts we faced numbers of deaths, but we didn't even have time to eat dinner or talk to others about it*.

#### The lack of emotional support in the work place

Due to the compressed work situation in hospitals and shifting working hours for people in different shifts there was no possibility of being alone and talking about emotions and feelings.

*35 year old /female:* ^*^ *There was no opportunity to talk to anyone at work, we constantly had to see critically ill patients and we had no energy left to talk*.

*38 year old /male:* ^*^ *Each of us was handling our own worries. Who could talk to others about their feelings in such situations?*

*45 year old /male:* ^*^ *We were exhausted of constantly talking about illness, death and all the hardships we were facing. I preferred not to talk about anything else other than work*.

#### Inability to express their emotional experience and sense of grief during loss

Some felt stunned and emotionally blocked. They attributed their numbness to their upbringing or personality types.

*38 year old /male:* ^*^It has nothing to do with COVID-19. It is difficult for me to talk about my emotions with others.

*45 year old /male:* ^*^ *Why should I go and tell others that I'm sad? I don't know how to do that. I'll do something about it myself. It's an embarrassment for me to do so*.

#### Exposure to COVID-19 patients and its high mortality rate

These people expressed that the rapid spread of the disease and the high mortality rate made them feel unsure about the future. It stopped people from being able to cope with what was happening.

*32 year old/female:* ^*^Our patients were dying so quickly that we couldn't understand what was happening.

*45 year old/female:* ^*^It only took a few days from the time my aunt told me that my uncle was sick until he died. He was healthy and had no history of medical illness. He was middle aged and he died suddenly. Now after several months, I still haven't been able to come to terms with it.

#### Not being able to hold funerals

They expressed that to be able to stop the illness from spreading they did not hold a ceremony or memorial and this was extremely difficult for them because in our culture it is important that people stand by each other during the first days of a loved one's death and help each other come to terms with it. In most Iranian families there is a strong interpersonal interaction and people are by each other's sides during happy and sad moments. However, because of the fear of losing more people during the COVID-19 pandemic many changes were made.

*35 year old /female:* ^*^Just the family came and the traditional ceremony was not held. They had a ceremony at the grave yard and another one with thirty people. However, I was more worried about my parents who are elderly. My mother was very close to my aunts and I was worried about her. I was scared that she would have a hard time at the memorial and I wanted to be by her side.

*38 year old /male:* ^*^In the beginning, we didn't have any official ceremonies because of COVID-19, but our relatives came themselves. We weren't able to hold a ceremony.

### Second theme: New experiences for coping with loss

People used different ways to cope with death and they chose different systems to heal. Many of the people who were interviewed used systems that came from their own spiritual culture to calm them down in such days.

#### Keeping themselves busy to avoid annoying thoughts and memories of the deceased

Some spent their time grieving the loss of their loved ones by constantly facing critically ill COVID-19 patients. They were trying to keep their minds occupied in different ways.

38 year old /male:^*^ *I tried to sleep during my free time to stop my mind from thinking about the death of my dear one*.

*35year old/ female:* ^*^I spent more time with my husband and children so I wouldn't be alone. Any time I was alone my mind started to think of death.

#### Taking time off

The duration of the time people needed off work to grieve their loved ones varied and some expressed that they needed more time off to be able to come to terms with the fact that their loved one was gone and others expressed that long working hours of not working made them stay home and do nothing. The memories of their deceased loved ones would keep on coming back to them and make these days miserable. It stopped them from going back to their normal routine. They wanted to work even if it meant returning to wards with critically ill patients busying themselves working.

*48 year old /female:* ^*^I try to keep my mind off. I try to take more shifts and spend more time at work to keep my mind off. If I'm alone, I spend all my time thinking.

*28 year old /female:* ^*^ *I wish I had more time off work to spend more time with my family while grieving and I wish I could stay in my home town to grieve*.

*39 year old /female:* ^*^ *I'm glad that I quickly came back to work, my co-workers are empathetic and I feel useful. I'm not alone*.

#### Changing to non-COVID-19 wards

Some expressed that dealing with critically ill COVID-19 patients made them review the memories of the last days of their loved ones. Therefore, they asked to change their working place and move to a ward where there were no COVID-19 patients.

*48 year old female:* ^*^I myself have cardiovascular problems and I couldn't stand facing critically ill COVID-19 patients. It was difficult. I wanted to go to other wards.

#### The role of religion and spirituality

Many of the interviewed people used religious methods to deal with grief. Strong cultural and religious beliefs in people helped them facilitate the experience of dealing with grief.

*28 year old /female:* ^*^ *I was raised in a religious family. After my father's death, I started to pray more often as if it calmed me down. I turned to God and this calmed me down*.

*35 year old/ female:* ^*^ *I still haven't completely dealt with it. The only thing that calms me down is that I tell myself that what happens is only God's will. Life and death are in God's hands*.

*38 year old /male:* ^*^ *I read the biographies of religious leaders. They had also gone through hardships in their lives. This made me follow them and accept death as a truth much more easily*.

#### Receiving and asking support from co-workers, family members, hospital and society

Among the people who were interviewed the women had more tendency to ask for and receive support from the people around them. They expressed that it was easier for them to ask for emotional help at work and at home. They received help to calm down their families.

*48 year old/female:* ^*^ *The group members we worked with were nice people. I would talk to them about my feelings and they would support me. I didn't feel alone. After work I would talk to my husband, and it helped me a lot*.

### Third theme: More empathy for patients

A lot of the people who were interviewed expressed that after the loss of a dear one, they felt more empathetic and compassionate toward patients. While helping them they would remember their recently lost loved one. They recalled how much pain they had suffered in the last days and this helped them concentrate and pay more attention to their patients and the people who were accompanying the patient.

#### The need to continue working in the COVID-19 ward

Some expressed that even though they had lost their loved one to COVID-19, they still had the desire to work in this ward.

*48 year old /female:* ^*^ *I am actually a positive person when it gets to hospitals no matter how often my co-workers say that it is difficult. There was a discussion among us regarding whether we would allow our daughters to become nurses or not. I was the only person who said why not if she wants to because nursing is an occupation in which you can be kind to the patients, especially in a time such as the COVID-19 pandemic. I don't regret working in this ward*.

*39 year old /female:* ^*^ *If for a moment you can imagine that the person you are responsible for is your father, mother, brother or a relative you can be kinder to them*.

#### More attention and kindness for patients

Dealing with COVID-19 patients caused people to be more empathetic and kinder.

*39 year old /female:* ^*^ *I would give patients water. I even paid attention to the way they looked at me when I was tired*.

*28 year old /female:* ^*^ *It didn't make any difference in the number of people who passed away but it helped us treat them much better while they were alive. I somehow felt that they needed more attention, especially those who felt worse. Those who felt better would come and get remdesivir and go but those who were sicker needed more attention. We would think that they might not be around the next night*.

#### More understanding of the family situation

The individuals who were interviewed expressed that because they had taken care of their loved ones who were ill before their death, and had experienced all that the person next to the patient was experiencing, they were able to be more empathetic at work.

#### Spending more time caring for the patients to decrease the feeling of guilt

Some say that because they weren't able to spend time with their loved one, they felt guilty and now they feel better by spending more time with the patients.

*28 year old /female:* ^*^ *My father's death affected my interactions with COVID-19 patients and made me more empathetic. My father's memories came back to me, especially because I work in the type of environment in which my father passed away*.

*48 year old/ female:* ^*^ *It affected me a lot. Making the decision to stay in the COVID-19 ward was easier. The situation which I had been in helped me talk better to the patients. In the beginning of COVID-19, unlike now, the patients didn't have anyone by their sides and we were the only people who were talking them. We were the only ones there to explain that for example you are going to go to the ICU, don't worry and we would explain what was about to happen. I felt that I could make up for all that I wasn't able to do for my father*.

### Fourth theme: Change the meaning of death

These people explained that during the COVID-19 pandemic, especially after the death of their loved ones, the concept of death changed for them.

#### Normalization of the concept of death and feeling apathetic

Some expressed that because they repeatedly faced death they became apathetic and numb toward it.

*28 year old /female:* ^*^ *I had become cold hearted*.

#### Accepting death as an inevitable phenomenon and having less fear

Due to the constant daily exposure of health care workers to the death of patients from COVID-19 and due to endless attempts to save patients, these workers gradually developed an acceptance of death for they watched life go on regardless of what was happening. In this way their fear of death grew less.

*39 year old /female:* ^*^ *Generally speaking, I have come to better terms with death. Perhaps in the past I was scared but I don't worry much about it anymore*.

*32 year old /male:* ^*^ *In facing the death of the first patients, we were terrified but we were numb toward the patient's death. We had become used to it*.

### Fifth theme: The need for support in work places

In crises the fact that others are thinking of you can have a great role in controlling the person's situation, a lot of colleagues were complaining that they were not receiving any support from the system they were working in.

#### The need to receive empathy from the work place at a time of grief

In times of crisis people fight to go on living while they are exhausted both physically and mentally. Being in an empathetic environment where they can feel loved and appreciated allows their ability to face and fight problems to increase. It gives them a sense of belonging and of being a member of a team. This can be an important factor in increasing resilience.

*39 year old/ female:* ^*^ *We weren't expecting to have more time off because there was a lack of work force but they could have at least sent us a message of condolences*.

*45 year old /male:* ^*^ *If you go and give my name to the head of the wards of the hospital they wouldn't know me. Not that I expect the manager of the hospital to know me, but how many people lost their families at this time? The least they could have done was to have expressed condolences. We are all actually working here and everyone is getting benefits. It's not that we just work to get paid, everyone gets benefits*.

*28 year old /female:* ^*^ *Perhaps it's not right but I keep on going over what happened. I agree that COVID-19 is a disease which doesn't have a specific cure. In the beginning they did some things such as plasmapheresis. I insisted they inject IVIG which my father did not tolerate and they didn't give it to him again. But they didn't accept to freeze the plasma. Perhaps I wasn't in a situation to give such suggestions but no one suggested it to my family. I don't know but maybe it could have saved my father*.

#### The need to increase personal protective supplies in crises

There was a considerable fear of getting infected, which in addition to discrimination on distribution of protective supplies led to desperation of health care workers, particularly at the beginning of the Pandemic.

*39 year old/ female:* ^*^ *In the beginning we didn't have suitable clothing. They wouldn't give N95 masks to the staff. The beginning was horrible*.

*38 year old /male:* ^*^ *We lacked personal protective facilities and we didn't receive much support from the hospital*.

#### The need to reduce working hours

They express that the hospital could have been more responsible, given more welfare amenities to the staff and reduced the working hours to avoid personnel exhaustion.

*39 year old /female:* ^*^ *We didn't receive much support from the hospital, they didn't give us any time off. On the third day, they called my sister telling her that I should return to work without asking if we had buried our loved one yet or not*.

*48 year old/ female:* ^*^ *It would have been better if they had given us time off work. But it wasn't possible at that time. We can't blame the hospital. It was the peak of COVID-19 and most of the staff were sick. The shift that I was working (the time my aunt died) was when my colleague was sick and I had to stand instead of him. The hospital could have shut down another ward to switch the staff to our ward but they didn't do that*.

## Discussion

Grief is a subject that requires utmost consideration in a global pandemic that took the lives of over 6 million people in just 2 years. There are some studies addressing grief in non-healthcare workers during the COVID-19 pandemic (29). However, the impact of personal loss (death of a loved one) and professional loss (death of patients) on the mental health of intensive care workers and other essential workers and how they grieve for these losses have not been well studied.

The experience of grief has often been challenging for ICU staff, even before the COVID-19 pandemic, and it has been more troublesome for those who lost loved ones. For example, there is a similarity in patient mortality rates between cancer patients and COVID-19 patients. Studies have shown that oncologists may experience a sense of failure or compassion fatigue (emotional and physical exhaustion that reduces their ability to empathize or feel compassion for others) after a patient's deaths (30, 31). As with health care workers caring for COVID-19 patients, oncologists witness their patient's suffering first hand and often feel responsible for their suffering and even for their deaths (32). Granek et al. found that oncologists experienced powerlessness (65%), self-doubt (60%), guilt (35%), failure (55%), sadness (70%), and loss of sleep (30%) when grieving for a deceased patient (31). However, the COVID-19 pandemic has heightened healthcare workers' fear and anxiety, aggravating pre-existing clinicians' “professional grieving” difficulties. Factors including a high mortality rate, being unprepared for the new conditions of work, absence of a cure, constantly changing treatment guidelines, demographics of patients, and sudden/unpredictable death of patients enhance the risk of ICU staff undergoing complicated or persistent grieving “I have become more cold-hearted than before“ is what we repeatedly heard from the interviewees in this study, and this may show compassion fatigue to some extent (28).

The ICU staff's loss and grief during the COVID-19 pandemic have become more personal than ever because illness and death are possible for them and their loved ones. There has always been a chance of ICU staff experiencing counter-transference with patients who remind them of a loved one, but the epidemic has intensified these correlations (33). “I felt more compassion toward a patients/patients' family” is a statement that most of our participants reported during our interviews. It seems that the ICU staff's perception of grief, patients' suffering and family members' hardships changed after they lost loved ones. Our own findings strongly support that view and show some important points including: complex grieving processes, new experiences for coping with loss, more empathy for patients, different meanings of death, and the need for the support of various workplaces.

However, it is unclear if their compassion is restored or if they are trying to compensate for any shortcomings toward deceased loved ones that they regret as one of our participants mentioned.

People experience grief differently in diverse cultures and contexts.

In Iran there is also several subcultures but generally continuous presence of people around survivors in early days of grief, is among important factors of effective social support. Consistent with the previous literature, the themes of this study showed that this effective social support has been reduced (8).

### Strengths and limitations

To the best of our knowledge, this is the first qualitative study on the experience of grief among ICU staff during the COVID-19 pandemic in Iran.

Our sample was limited due to the fact that participants were self-selecting and came from one hospital and our findings may represent the experiences of this group thus limiting wider generalizability.

### Implication of practice, research and policy

It is critical to pay particular attention and to support those working in stressful, sensitive positions such as ICU personnel who have suffered grief because it can directly affect the quality of their work. Quantitative tools must be prepared and used for the study of larger samples in different cultures.

For future researchers, we suggest conducting studies on a wider range and promoting comparisons between different ICU staff in different hospital care units to improve the quality of research, and we recommend an organization be set up to identify and support at-risk staff so that a supportive intervention can be built.

Much study is needed to better understand professional grief processes in critical care workers, risk factors for complicated grief, and how to help them persevere during the crisis. Managers of health systems must invest in policies based on wellbeing and functionality.

## Conclusion

Profound loss and enduring grief were described by ICU staff who lost their loved ones during pandemic circumstances.

Different healthcare workers have different experiences when they face crisis and loss in their lives. Their cultural, spiritual, personal and work backgrounds have great effects in the formation of the way they handle crisis.

Health care professionals, especially those who worked in the ICU, experienced various degrees of stress and pain depending on their personalities, defense mechanisms, subcultures, family situations, career and work place during the COVID-19 pandemic. If we ignore them, they will remain hidden sufferers and this will have negative effects on the quality of their lives and their wellbeing.

Paying attention to the details of the ICU staff's life, gender differences, cultural and spiritual aspects and assessing all the angles of people's lives can give us a better understanding and perception of their experiences during crisis and loss. This understanding can bring out some valuable points which can help policy makers pass better laws for the wellbeing of society and people so as to promote leadership in turbulent times.

## Data availability statement

The raw data supporting the conclusions of this article will be made available by the authors, without undue reservation.

## Ethics statement

The studies involving human participants were reviewed and this research was approved by the Ethics Research Committee of the Iran University of Medial Sciences with unique number IR.IUMS.REC.1400.1148 and protocol number 22923. The patients/participants provided their written informed consent to participate in this study.

## Author contributions

SN: project ideation, research design, interviews, supervision, analysis, article writing, and coordination. MA: research design, supervision, and article writing. MS and HM: article writing and interviews. LG: analysis, coordination, and supervision. All authors contributed to the study design and article writing. All authors contributed to the article and approved the submitted version.

## Conflict of interest

The authors declare that the research was conducted in the absence of any commercial or financial relationships that could be construed as a potential conflict of interest.

## Publisher's note

All claims expressed in this article are solely those of the authors and do not necessarily represent those of their affiliated organizations, or those of the publisher, the editors and the reviewers. Any product that may be evaluated in this article, or claim that may be made by its manufacturer, is not guaranteed or endorsed by the publisher.
